# Functional Analysis LRP6 Novel Mutations in Patients with Coronary Artery Disease

**DOI:** 10.1371/journal.pone.0084345

**Published:** 2014-01-10

**Authors:** Yujun Xu, Wei Gong, Jia Peng, Haoran Wang, Jin Huang, Hu Ding, Dao Wen Wang

**Affiliations:** 1 The Institute of Hypertension and Department of Internal Medicine, Tongji Hospital, Tongji Medical College, Huazhong University of Science and Technology, Wuhan, China; 2 Intensive Care Unit, West China Hospital, Sichuan University, Chengdu, China; 3 Echocardiography Laboratory, Sichuan Provincial Hospital, Chengdu, China; 4 Genetic Diagnosis Center, Tongji Hospital, Tongji Medical College, Huazhong University of Science and Technology, Wuhan, China; FuWai hospital, Chinese Academy of Medical Sciences, China

## Abstract

**Background:**

Genetic architecture of coronary artery disease (CAD) is still to be defined. Since low density lipoprotein receptor-related protein 6 (LRP6) gene play critical roles in Wnt signal transduction which are important for vascular development and endodermis specification, we therefore resequenced it to search for mutations in CAD patients.

**Methods:**

We systemically sequenced all the exons and promoter region of LRP6 gene in a sample of 380 early onset CAD patients and 380 control subjects in Chinese.

**Results:**

In total, we identified 5 patient-specific mutations including K82N (two patients), S488Y (one patient), P1066T (two patients), P1206H (two patients) and I1264V (one patient) All these mutations located at the extracellular domain of LRP6 gene. In vitro functional analysis of patient-specific mutations demonstrated that these mutations resulted in a significant reduction in both protein level transporting to cell membrane and downstream Wnt signal activity. Furthermore, we found that LRP6 novel mutations attenuated proliferation and migration of human umbilical vein endothelial cells (HUVECs) when compared with wild type (WT) LRP6.

**Conclusion:**

Our results demonstrated that these loss-of-function variants might contribute to disease liability in a subset of CAD and defects in Wnt signal activation might be important contributing factors for the onset of CAD.

## Introduction

Coronary artery disease (CAD) arising from atherosclerosis and its consequences such as myocardial infarction (MI), arrhythmias and heart failure are the leading causes of death and morbidity worldwide [1]. Affected individuals cluster in families in patterns that reflect the sharing of numerous susceptibility genes [2]. Genome wide association studies, involving tens of thousands of cases and controls, have mapped common disease variants to many distinct loci. However, the common variants explain 4% of inter-individual variation in disease risk and no more than 13% of the total heritability of coronary disease [3]. It is necessary to search for rare genetic variants (or mutations) in candidate gene studies by deep sequencing which could provide potentially novel direction for research into pathogenesis of this disease [4].

Specifically, previous studies indicate that Wnt signaling pathway is involved in the pathogenesis of atherosclerosis in some key stages of atherosclerotic plaque formation such as endothelial dysfunction, intimal thickening, inflammation, foam cell formation, calcification and angiogenesis [5]–[9]. Among genes involved in Wnt signaling, LRP6 has been studied for its role in initiating β-catenin-dependent pathway. Reduced LRP6 expression levels in carotid atherosclerotic lesions led to retarded Wnt signaling which may contribute to atherosclerosis development [10]. In addition, LRP6 mutation in a family could lead to hypertension, type 2 diabetes and early CAD [11]. Also this mutation in LRP6 could increase PDGF-dependent vascular smooth muscle cell proliferation, which contributes to development of early atherosclerosis in humans [12]. However, whether other mutations located in LRP6 could contribute to the development of CAD still to be identified.

To further investigate the possible role of LRP6 alterations as well as Wnt signaling in the pathogenesis of CAD, we screened the entire coding region of LRP6 gene in 380 early onset CAD patients and 380 control subjects. As a result, we identified five patient-specific point mutations. Importantly, functional analysis revealed that these variants were loss-of-function mutations which might contribute to the development of CAD in some Chinese.

## Methods

### Subjects

A total of 380 cases were selected from among early onset CAD patients undergoing coronary angiography within the Tongji Hospital and the Institute of Hypertension (Wuhan, China) between October 2007 and September 2011. All cases underwent coronary angiography and early-onset CAD was defined as significant stenosis (>50%) of at least one major epicardial vessel at ≤55 years in men and ≤60 years in women. Subjects with congenital heart disease, cardiomyopathy, valvular disease, and renal or hepatic disease were excluded from the study. Three hundred eighty ethnically and geographically matched controls, who had not suffered from cardiovascular disease, were randomly selected from healthy residents in the community following the same exclusion criteria as cases. Patients and controls were interviewed to collect information on demographic characteristics, medical history, and behavioral habits. Risk factor prevalence was determined by physician diagnosis and/or treatment for hypertension, hyperlipidaemia, and diabetes. Smoking was defined as a history of smoking >2 pack-years and/or smoking within the preceding 1 year. The study was approved by the Tongji Institution Review Board and written informed consent was obtained after the procedures were fully explained.

### LRP6 gene mutation screening

Genomic DNA was prepared from peripheral blood cells using the QG-Mini80 workflow with a DB-S kit (FUJIFILM Corporation, Tokyo, Japan) according to the manufacturer's instructions. DNA was quantified and diluted to a final concentration of 10 ng/μl.

Optimal PCR primer sequences were generated to amplify all the exons and the putative promoter region of the LRP6 gene. Primer sequences, optimal annealing temperatures and size of each amplicon are listed in Table S1 in File S1. PCR products were then purified to remove residual primers and dNTPs and eventually sequenced using the BigDye Teminator Kit (Applied Biosystem, Foster City, CA, USA) and analyzed on the ABI Prism 3130 xl automated sequencer (Applied Biosystem, Foster City, CA, USA). The chromas program (Technelysium Pty. Ltd., Helensvale, Queensland, Australia) was used to identify mutation candidates that were then confirmed by two independent observers. All the variants were verified by repeated PCR and sequencing in both directions.

Predictions of potential deleterious effects of missense mutations detected in the LRP6 gene was performed using the software tools PolyPhen-2 (http://genetics.bwh.harvard.edu/pph2/) and SIFT (http://sift.jcvi.org/). Multiple sequence alignment was finished by using Cluster X program[13].

### Luciferase assays and Western-blot analysis of LRP6

The Wnt/beta-catenin pathway is activated when a Wnt ligand binds to the Frizzled receptor and its coreceptor (LRP6). The formation of a likely Wnt-Fz-LRP6 complex and subsequent events lead to inhibition of the Axin-mediated beta-catinin phosphorylation and to the stabilization of beta-catenin, which travels to the nucleus to form complexes with LEF and activates Wnt target gene expression [14]. We subsequently used a well characterized luciferase signaling system to examine the effect of patient-specific mutant LRP6 on Wnt signaling [15]–[16]. In brief, we transiently transfected plasmids encoding LEF-1, reporter gene (which has LEF-1-responsive promoter fused to the luciferase gene), C-terminal HA-tagged LRP6 (wild-type or mutant) and an inner control plasmid pRL-TK in the HEK293T cell line together.

Plasmid pcDNA3.1 containing human wild type LRP6, which was modified with a hemagglutinin (HA) tag at the C terminus, was kindly provided by Moon [17]. Site-directed mutagenesis method was used to generate LRP6 mutants as Zheng described [18]. The WT sequence and the presence of mutations were confirmed by sequence analysis. Overlapping primers for each mutagenesis were listed in Table S2 in File S1.

Human embryonic kidney (HEK) 293 T cells were cultured as previously described [19]. Twenty-four hours prior to transfection, HEK293 T cells were plated in 24-well plates at 2.5×10^5^ cells per well. Plasmids encoding LEF-1 (a transcription factor activated by Wnt signal) constitutively expressed from a cytomegalovirus promoter, luciferase under control of an LEF-1-responsive promoter, and pRL-TK (an inner control plasmid expressing Renilla reniformis luciferase) were introduced by transfection into cells seeds in 24-well plates, using Lipofectamine™ 2000 transfection reagent (Invitrogen, Carlsbad, CA) according to the manufacturer's instructions. In addition, plasmids encoding wild-type LRP6 or mutants and plasmid expressing Wnt-1 were transfected in indicated combinations (Table S3in File S1). The total DNA concentration in each transfection experiment was kept constant (1 µg per well) by adding varying amounts of empty vector (pcDNA3.1) if necessary. After 24 h, cells were lysed and luciferase reporter activity was measured in duplicate by using 100 µl of lysate in accordance with the dual luciferase assay specifications (Promega Madison, WI USA). Typically, each experiment was repeated in at least three different batches of cultures.

Mem-PER Eukaryotic membrane protein extraction reagent kit (PIERCE, Rockford, IL USA) was used to enrich membrane protein from cultured cells. Antibody for HA tag was purchased from Sigma-Aldrich. Antibodies for GAPDH,β-actin and α-tubulin was purchased from Santa Cruz Biotechnology. Wild type LRP6 or variant constructs were transfected in HEK293 cells as described above. Twenty-four hours after transfection, cell lysates were prepared as previously described [20], and then were separated by SDS-polyacrynamide gels (10%) and transferred to polyvinylidene difluoride membranes (Bio-Rad, Hercules, CA). The membranes were blocked with 5% nonfat dry milk in TBS-T (10 Mm Tris-Cl (pH 8.0), 100 mM NaCl and 0.1% Tween 20) for 2 hours. After that the lanes were incubated overnight with specific antibody, followed by incubated with secondary antibody for 2–3 hours. Proteins were visualized by enhanced chemiluminescence according to manufacturer's instructions (PIERCE, Rockford, IL USA). All groups were then normalized to their respective controls, and bar graphs represent quantification of at least three independent experiments.

### Cell Migration

HUVECs were transfected and exposed to RPMI 1640 with 10% FBS for 48 h and then cell migration was assayed using Transwell chambers technique [21]. Briefly, The HUVECs transfectant cells were detached from the culture plates and replated onto the inserts of 8-µm pore sized Costar Transwell chambers (Corning Inc., Corning, NY, USA). The cells that migrated to the lower surface of the filters were fixed in methanol and stained with crystal violet. Cells on the lower surface of the membranes were counted.

### Cell Proliferation Assay

Assessment of cell viability was performed using the Cell Counting Kit-8 (CCK-8) assay. The HUVECs transfectant cells were incubated in 10% CCK-8 (Beyotime Institute of Biotechnology, Nantong, China) diluted in normal culture medium for 2 hour at 37°C in 96-well culture plates. The number of viable cells was assessed by measurement of absorbance at 450 nm for each well using a microplate reader (Bio-Tek Instruments, Inc., Winooski, VT, USA) according to the manufacturer's instructions.

### Statistical analysis

Statistical analysis was performed with SPSS 13.0 (SPSS Inc, Chicago) for Windows (Microsoft Corp, Redmond, Wash). All quantitative variables were generally described as means with standard deviation (SD). The distribution of quantitative variables was tested for normality by use of a 1-sample Kolmogorov-Smirnov test. For comparison of the baseline characteristics among groups of subjects, quantitative variables, including age, body mass index, LDL-C, TG, FPG and TC, were compared with 1-way analysis of variance and Dunnett's test. A chi-square (χ2) test was used to compare qualitative variables. All quantitative studies about cell migration and proliferation were performed at least in triplicate, with the results expressed as means ± SD as appropriate. Statistical significance for graphs of luciferase assay, cell migration and proliferation were performed by one-way ANOVA followed by post hoc t tests using Prism v4 (Graphpad Software Inc). A p value <0.05 was considered statistically significant (two-tailed).

## Results

### General Characteristics of the Subjects

Demographic features of the subjects in our study were shown in Table 1. Ages of the study population were 50.3±7.7 (mean±SD) years in CAD cases and 55.3±8.8 years in control individuals (p<0.010). As expected, the traditional CAD risk factors such as hypertension, diabetes, BMI, smoking status were significantly different between the cases and controls. However, LDL-C and total cholesterol levels were significantly lower in cases than in controls, which could be the result of cholesterol-lowering medication in the patients after diagnosis. The proportion of subjects reported taking cholesterol-lowering medications such as a statin in the cases and controls in our study were 93.4% and 1.3%, respectively.

**Table 1:** Clinical characteristics of the subjects studied.

	ECAD (n = 380)	Control (n = 380)	*p* value
male (%)	73.2	72.7	0.87
age (years)	50.3±7.7	55.3±8.8	<0.01
BMI	24.9±3.3	24.4±3.3	<0.05
Hypertension (%)	52.9	25.0	<0.01
Diabetes (%)	16.9	6.0	<0.01
Smoking (%)	61.6	45.6	<0.01
LDL-C (mmol/L)	2.53±0.96	2.85±0.73	<0.01
TG (mmol/L)	2.03±1.43	1.37±1.28	<0.01
FPG (mmol/L)	6.89±2.72	4.87±1.20	<0.01
TC (mmol/L)	4.51±1.17	4.76±0.88	<0.01

ECAD, early-onset coronary artery disease; BMI, body mass index; LDL-C, low-density lipoprotein cholesterol; TG, triglyceride; FPG, fasting plasma glucose; TC, total cholesterol. Data presented as mean±standard deviation for age, BMI, LDL-C, TG, FPG, TC or number (%) of patients for other characteristics.

### Identification of missense mutations of LRP6 gene

**Figure 1:**
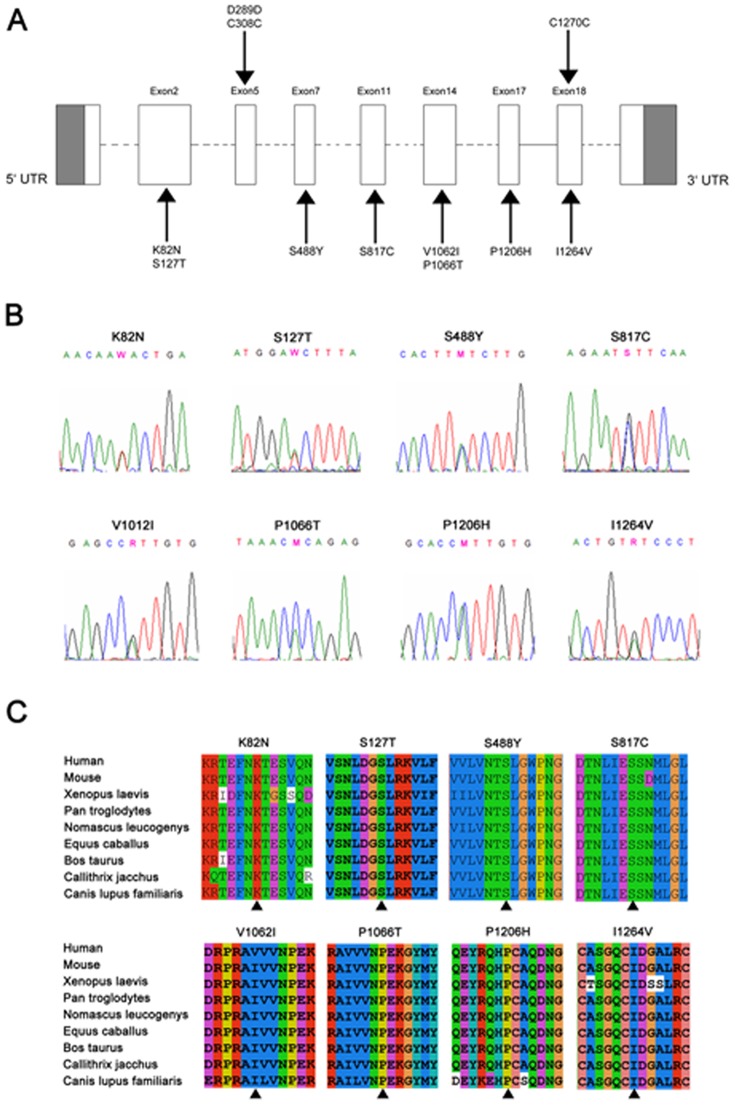
Genomic structure of the exons encoding the open reading frame of LRP6 and identified mutations or polymorphisms. **A** Distribution of the 11 missense mutations or polymorphisms found in this study. Variants resulting in amino acid changes were listed below gene map, and those did not result in amino acid changes were indicated above gene map. **B** Sequence electropherograms of eight missense mutations or polymorphisms identified in this study. **C** LRP6 peptide sequences surrounding the mutated residues with multiple interspecies alignments generated by ClustalX. The mutation position is indicated with an arrow.

Following the systemic sequencing of all the exons and the promoter region of LRP6 gene in 380 early onset CAD patients and 380 control subjects, we totally identified 11 genetic variants (three synonymous and eight missense) (Table 2 and Figure 1A). All the sequencing data were shown in Figure 1B. Notably, among these 11 variants, 5 novel missense mutations were found limited to case group, including a A-to-T substitution (c.388A > T) at codon 82 (K82N) in two early onset CAD patients; a C-to-A substitution (c.1605C > A) at codon 488 (S488Y) in one patient; a C-to-A substitution (c.3338C > A) at codon 1066 (P1066T) in two patients; a C-to-A substitution (c.3759C > A) at codon 1206 (P1206H) in two patients; a A-to-G substitution (c.3932A > G) at codon 1264 (I1264V) in one patient. Clinical characteristics of these novel mutation carriers were shown in Table 3. In total, eight mutations were identified in 380 early CAD patients, accounting for 2.1% of this patient population. And we did not identify any CAD patient carried with multiple mutations. However, no novel mutation was identified in control group.

**Table 2:** Genotype and allele frequencies of the identified variants in the patient and control group.

Location	SNP ID	Function	Group	Allele (1/2)	Genotype counts			MAF(%)
					1/1	1/2	2/2	
exon 2	c.388A>T	nonsynonymous	case	A/T	378	2	0	0.26
		K82N	control		380	0	0	0.00
	c.521T>A	nonsynonymous	case	T>A	368	12	0	1.58
	(rs17848270)	S127T	control		371	9	0	1.18
exon 5	c.1009C>T	synonymous	case	C>T	377	3	0	0.39
		D289D	control		378	2	0	0.26
	c.1066T>C	synonymous	case	T>C	377	3	0	0.39
		C308C	control		377	3	0	0.39
exon 7	c.1605C>A	nonsynonymous	case	C>A	379	1	0	0.13
		S488Y	control		380	0	0	0.00
exon 11	c.2592C>G	nonsynonymous	case	C>G	374	6	0	0.79
	(rs2302686)	S817C	control		374	6	0	0.79
exon 14	c.3326G>A	nonsynonymous	case	G>A	328	50	2	7.11
	(rs2302685)	V1062I	control		331	46	3	6.84
	c.3338C>A	nonsynonymous	case	C>A	378	2	0	0.26
		P1066T	control		380	0	0	0.00
exon 17	c.3759C>A	nonsynonymous	case	C>A	378	2	0	0.26
		P1206H	control		380	0	0	0.00
exon 18	c.3932A>G	nonsynonymous	case	A>G	379	1	0	0.13
		I1264V	control		380	0	0	0.00
	c.3952C>T	synonymous	case	C>T	373	7	0	0.92
	(rs1012672)	C1270C	control		375	5	0	0.66

MAF, minor allele frequency.

**Table 3:** Clinical characteristics of novel mutations carriers.

Individual ID	Mutation	Sex	Age at diagnosis	BMI	FPG	TG (mmol/L)	TC (mmol/L)	HDL (mmol/L)	LDL (mmol/L)
DP0539	K82N	male	46	30.06	7.85	4.34	4.69	0.72	2.08
DP0703	K82N	male	52	22.86	5.25	3.90	4.43	0.84	2.48
DP0426	S488Y	male	51	22.49	7.44	1.62	6.01	0.96	4.07
DP0007	P1066T	male	39	21.61	7.77	1.18	4.14	1.12	2.20
DP0968	P1066T	male	55	23.31	5.96	1.62	3.41	1.06	1.63
DP0999	P1206H	male	55	28.69	5.75	2.50	4.71	1.02	3.04
DP1201	P1206H	male	54	23.74	18.33	0.64	4.43	0.97	3.24
DP0578	I1264V	male	45	23.66	5.30	0.41	3.70	1.08	2.06

BMI, body mass index; FPG, fasting plasma glucose; TG, triglyceride; TC, total cholesterol; HDL, high-density lipoprotein cholesterol; LDL-C, low-density lipoprotein cholesterol.

### *In silico* analysis of LRP6 gene variants

Comparative amino acid sequence analysis revealed complete conservation of the amino acid sequence of LRP6 in nine species (human, mouse, Xenopus laevis, Pan troglodytes, Nomascus leucogenys, Equus caballus, Bos Taurus, Callithrix jacchus, Canis lupus familiaris) at each of these positions except for V1062I (Figure 1C and Table 4). In addition, all these nosynonymous mutations were located at the extracellular domain of LRP6.

**Table 4:** In silico analysis of five novel missense mutations of the LRP6 gene identified in this study.

Mutation in cDNA	Amino acid change	Predicted deleterious	
		PolyPhen-2^a^	SIFT^b^
A388T	K82N	benign	Tolerated
C1605A	S488Y	benign	Affect protein function
C3338A	P1066T	possibly damaging	Affect protein function
C3759A	P1206H	probably damaging	Tolerated
A3932G	I1264V	probably damaging	Affect protein function

^a^ PolyPhen-2: http://genetics.bwh.harvard.edu/pph2/;

^b^ SIFT: http://sift.jcvi.org/.

### Membrane expression of the mutant LRP6 is reduced

**Figure 2:**
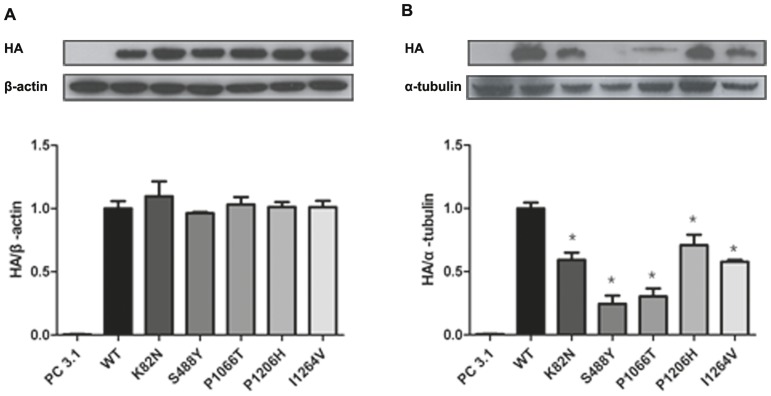
Expression levels of wild-type LRP6 and mutant LRP6 in HEK293T cell. **A** Expression of wild-type LRP6 and mutant LRP6 was no difference in the total expression levels. **B** Without the presence of Wnt-1 stimulation, mutant LRP6 constructs showed significant reduction in cell surface expression compare to wild type. WT, wild-type. *P<0.05 vs. WT.

Next, we carried out functional characterization studies for these five mutants. To evaluate the effect of the mutations on protein synthesis and membrane trafficking, WT and mutant LRP6 protein tagged with the hemagglutinin epitope sequence at the carboxy terminus were expressed in HEK293T cells. Equivalent amounts of wild-type LRP6 and mutant LRP6 were detected by western blot analysis in the total cell lysates, suggesting no difference in the total expression levels between wild-type and mutants (Figure 2A). However, in parallel experiments, mutant LRP6 constructs resulted in a significant reduction in protein level trafficking to cell surface compared to wild type while still harboring a similar level of total protein expression (Figure 2B).

### Wnt signaling is activated by mutant LRP6 to a minor extent

**Figure 3:**
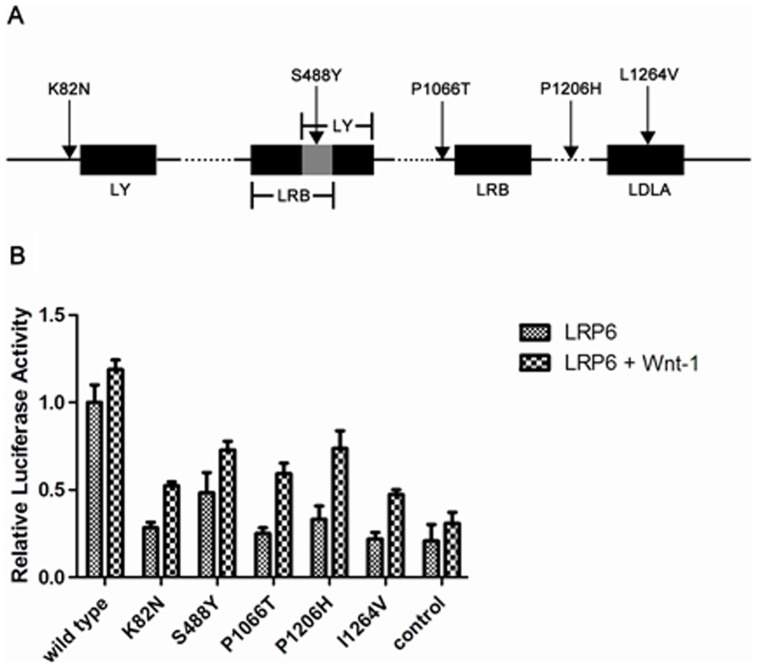
Location of LRP6 mutations and their effect on Wnt signaling. **A** The location of the de novo mutations in LRP6 are shown relative to the key protein domains. De novo mutations are indicated by black triangles. Black box indicates protein domains; gray box represents shared sequence of the overlapping protein domain. The following abbreviation is used: LY, low-density lipoprotein-receptor YWTD domain; LDLa, low density lipoprotein receptor class A domain; LRB, low-density lipoprotein receptor repeat class B. **B** Wnt signal is activated by mutant LRP6 to a minor extent. HEK293 cells in 24-well plates were transfected with LEF-1 expression plasmid, LEF-1 responsive luciferase reporter plasmid, LRP6 expression plasmid and Renilla reniformis luciferase expression plasmid in the presence or absence of 0.02 µg Wnt-1 expression plasmid followed by an assay of Wnt signaling as method indicated. The luciferase activities presented were normalized against the levels of Renilla reniformis. The results are shown as mean and standard error of the mean of triplicate experiments.

We transiently transfected plasmids in the HEK293T cell line as we described in method section. In the absence of Wnt-1, LRP6 mutants showed at least 50% reduction of induced signaling compared with that of wild-type LRP6 (all p<0.05). Furthermore, the addition of low doses of Wnt-1 still did not reverse the reduction of luciferase signaling (41% to 59% reduction, all p<0.05) (Figure 3).

### Effects of WT and Mutant LRP6 on HUVECs proliferation and migration

**Figure 4:**
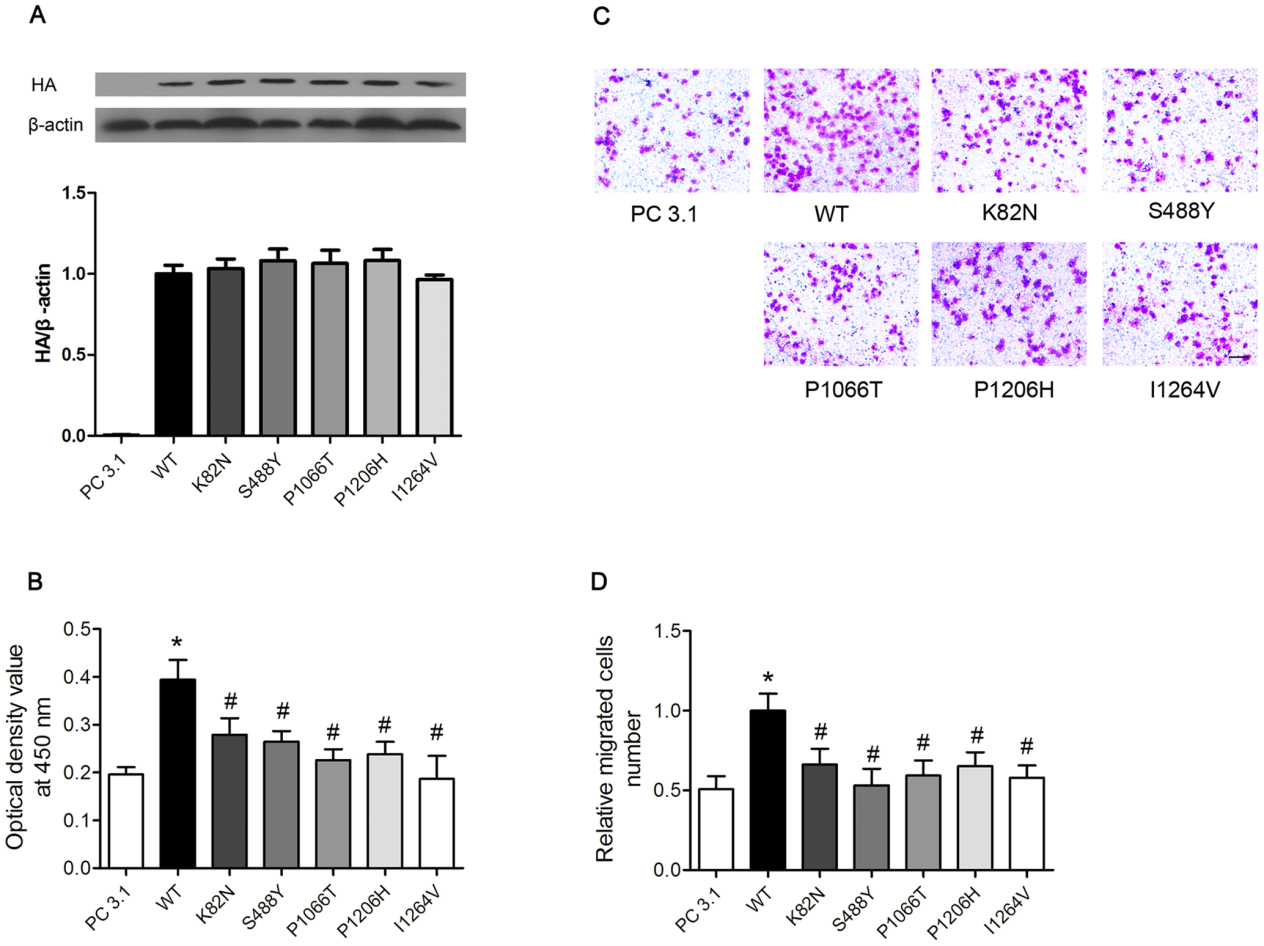
Mutant LRP6 reduce ability to enhance cellular proliferation and migration. **A** Expression of wild-type LRP6 and mutant LRP6 was no difference in the total expression levels in HUVECs cells. **B** In CCK8 assay, overexpression of wild type LRP6 stimulated HUVECs proliferation, whereas overexpression of LRP6 mutant stimulated cell proliferation to a minor extent. **C and D** Transwell chamber assay shows that the ability of enhancing HUVECs cells migration was reduced in mutant group compared with wild type. WT, wild-type. *P<0.05 vs. PC 3.1, ^#^P<0.05 vs. WT group.

We further examined the effects of transfection either with mutant LRP6 or wild type on cell proliferation and migration in HUVECs. Firstly, we detected LRP6 expression, and found no difference LRP6 expression in the total cell lysates levels between wild-type and mutants (Figure 4A). Result about cell proliferation assay in HUVECs was showed in figure 4B. Overexpression of wild type LRP6 stimulated HUVECs proliferation, whereas overexpression of LRP6 mutant displayed reduced effect (30% to 52% reduction, all p<0.05). Figures 4C and 4D shows the representative result of migration assay in HUVECs. LRP6 mutants showed reduction of migrated cell number compared with that of wild-type LRP6 (35% to 48% reduction, all p<0.05).

## Discussion

In this study, we identified five novel mutations in LRP6, which were detected in early-onset CAD patients only, no novel mutation was identified in 380 control individuals. Functional studies indicate that all five mutations exhibited impaired function in potentiating Wnt signal transduction, suggesting that they might be associated with CAD.

Wnt proteins can signal in a variety of ways, depending on the cell type and the receptors and/or co-receptors expressed [22]. LRP6, is a member of the LDLR family, which has the unique structure and function as an essential co-receptor for Wnt/β-catenin signaling [23]. Recent reports underline the important role of the Wnt system in vascular morphogenesis development, including cell fate specification, proliferation, and survival, and may use different receptors and signaling pathways [24]. In addition, there was mounting evidence for an involvement of the Wnt pathways in multiple processes involved in atherogenesis [5]-[6], [22], [25]-[26]. Among genes and signal molecules involved in the Wnt pathway, LRP6 is known to function as a indispensable co-receptor with the protein frizzled to bind extracellular Wnt glycoproteins which activate the canonical Wnt/β-catenin signaling pathway[27]. In accordance with these studies, our luciferase finding supported the concept that mutant LRP6, which identified in individuals with CAD, were much less effective in activating Wnt signaling.

Endothelial dysfunction was regarded as an early hallmark of atherosclerosis [28]. To achieve atherosclerotic regression, dysfunctional endothelial cells must be returned to basal homeostasis and dead cells need to be replaced. Local endothelial cell proliferation and migration is one of the known mechanisms that can result in endothelial cell replacement [29]. We pursued further experiments assessing whether LRP6 could affect endothelial cells function in the pathogenesis of atherosclerosis. And we found that mutant LRP6 stimulate endothelial cells proliferation and migration to minor extent compared with wild type, suggesting individuals with these mutation could suffer from atherosclerosis more easily as their ability to repair denuded vessel wall reduced.

Although LRP6 have been involved in human CAD, LRP6 mutations have not been investigated in large studies of patients with CAD. Functional analyses of the patient-specific mutations indicate a decrease in signal transduction, which is caused by their deficiency in trafficking from endoplasmic reticulum to the cell surface. And these mutant LRP6 were much less effective in enhancing cellular proliferation and migration. However, the five carriers with novel mutations had different phenotypes while no clear genotype-phenotype correlation could be established. As CAD is a complex genetic disease resulting from the interaction between environmental factors and specific susceptibility genes, it shows wide variability with respect to age of onset and severity of the disease. We suggested that the observed mutations may served as predisposing factors for CAD, but disease develop only once a critical threshold of liability is crossed, due to the cumulative contribution of environmental agents or of other genes.

Interestingly, early study has shown that the V1062I polymorphism could decrease Wnt/β-catenin signaling [17]. It is tempting to speculate that this variant is associated with atherosclerosis. Additionally, R.Sarzani also suggested this variant was an independent risk factor for carotid artery atherosclerosis in hypertensive patients [10]. However, we did not identify the association between this common polymorphism and early-onset CAD. Although limited by small sample size, this finding seems plausible as none of the genome wide association studies of CAD, including those performed mainly in Chinese Han population and had enough power, revealed V1062I (rs2302685) was associated with CAD. We hypothesize that the healthy phenotype of V1062I carriers in control group could be the result of an incomplete penetrance. The penetrance of V1062I variant may vary as genetic and environmental modifiers may have major effects in some pedigrees.

In conclusion, our discovery of rare LRP6 mutants and functional activity assays suggested that impairment of Wnt pathway activity may play a role in the development of CAD. We hypothesize that these patient specific variants represent a predisposing factor for a subset of CAD. Further studies are needed at both genetic and functional level to confirm this hypothesis. Our finding might be helpful to identify subgroup of patients for whom dedicated therapeutic strategies could be developed.

## Supporting Information

File S1 **Supporting tables.** Table S1, Sequences of PCR primers used for amplification and sequencing of human LRP6 gene. Table S2, Sequences of primers used for site-directed mutagenesis. Table S3, Plasmid amounts used in the transfection (ng).
(DOC)
