# Supplementary material for: Functional Analysis LRP6 Novel Mutations in Patients with Coronary Artery Disease
Source: PLoS One. 2014 Jan 10;9(1):e84345. doi: 10.1371/journal.pone.0084345 (PMC3888387; doi:10.1371/journal.pone.0084345)
Supplement: File S1 — Supporting tables. Table S1, Sequences of PCR primers used for amplification and sequencing of human LRP6 gene. Table S2, Sequences of primers used for site-directed mutagenesis. Table S3, Plasmid amounts used in the transfection (ng). (DOC) [file pone.0084345.s001.doc]

| **Table S1. Sequences of PCR primers used for amplification and sequencing of human LRP6 gene.** | | | | |
| --- | --- | --- | --- | --- |
| Region | Forward primer (5'→3') | Reverse primer (5'→3') | Size | Tm (^o^C) |
| P1 | AGGAAGCCAGAATACAAAACTCACT | CCCTCCCCTCCCCCTAGACACAT | 1861bp | 60 |
| P2 | GTTTTGCAAATAGGACCATGTGA | GGGGAAAGGCAGGTTTTGTTA | 720bp | 58 |
| E2 | CACTTAAACCGATTTGAAACGC | GGGTCAGGGTGGTGTATGTCA | 556bp | 56 |
| E3 | GCGGCCTGAGCTTTCTTTAATA | ATGCTACCCCTGGTGGAGAAC | 559bp | 58 |
| E4 | TGGGGCTTTTGGACACCTT | TCAGCCTCCCAAGTAGGATTTT | 547bp | 58 |
| E5 | CCTGTCTTTGCAGATGGTTTGAT | GATGCCACAGAACAAAACACTCC | 446bp | 60 |
| E6 | AGTTAGCCATTAAACAGCTTGACC | CAACACCCAAAGTCAGCAAACA | 851bp | 58 |
| E7 | CCCCTTCCTTGGCATTAACTG | GATCAGCAGCCATTTCTCATACC | 519bp | 58 |
| E8 | GGGGGAAAAGTGGTCAAATAGA | GTCAAACAATGAGGGAGGTGG | 1326bp | 56 |
| E9 | TTGGGAGCAAGACATAATCATAGG | GGTTTTTGTTGAACTCTGCCTG | 534bp | 56 |
| E10 | CTTGGCACTTCTGGATCCTCTT | CCATTCCCCTCTTTCTTCACCT | 568bp | 58 |
| E11 | TGTATTGGACTGAATGGGGTGG | AAACAGGATACAATTCCAGAACAGA | 324bp | 58 |
| E12 | GGTCAGAAGATAGATTGATTTCAGAGA | GAAACAAATAACCCCCTCTGGAT | 646bp | 58 |
| E13 | GTCCTCAGCCTTAGCTCTTCAAC | AAGCTACCAGGTCCAGAATTTCA | 474bp | 56 |
| E14 | AAAGAAAGTCTGGGCAGTGAAATC | CCCATGTAGGTGTAAGCATTGTG | 494bp | 56 |
| E15 | AGCCTTCTCAGCTGTGTGCC | GGGCAGTGAAATCAGAGAAAGTGT | 501bp | 60 |
| E16 | GCTCCTCCAGAGAAGTGCCAT | AAGATCGTGACACTGCACTCCA | 743bp | 56 |
| E17 | GGGTTGTCCCTTTTTCTCTTCTC | ATACAAATGAATGGAACACACGC | 347bp | 56 |
| E18 | TTGCGGGCAAAAGTTTATAGTG | ACCCCAACTGACACTAAGCCAA | 541bp | 56 |
| E19 | TTCTTGCCAGAGTTTGACTTTACATG | ACGCCCGGCTGATTTCTAT | 483bp | 56 |
| E20 | GTCTTCAGGGCGTGGTATGTATG | CAAACCATATCTAAGGCCTTCTGTG | 588bp | 58 |
| E21-22 | GTTCTAGGCCAGCTCTTTCAATG | CCTGAAAGGAAAGGATTTGTGG | 1250bp | 58 |
| E23 | TGAAAATTGCCTCTTGGTCTGTG | ATCTCCAGTATTCCCTACCCCAT | 594bp | 60 |
| P1-SF1^a^ | ATCTGATGGCTCCATTAGGGTT |  | —— | —— |
| P1-SF2^a^ | GATTTCCAACCCCGAAGACG |  | —— | —— |
| E6-SR1^a^ | CATCCAAGGCAAAAAACTAGCA |  | —— | —— |
| P, promoter region; E, exon region; SF, forward sequencing primer; SR, reverse sequencing primer. ^a^primer designed for better sequencing. | | | | |
|

| **Table S2. Sequences of primers used for site-directed mutagenesis.** | | |
| --- | --- | --- |
| Primer | Sequences (5’ to 3’ | Mutation |
| m388F | CGAACAGAATTTAACAATACTGAGAGTGTGCAGAATG | K82N |
| m388R | CATTCTGCACACTCTCAGTATTGTTAAATTCTGTTCG |  |
| m1605F | GTAGTATTGGTTAACACTTATCTTGGTTGGCCAAATG | S488Y |
| m1605R | CATTTGGCCAACCAAGATAAGTGTTAACCAATACTAC |  |
| m3338F | AGCCGTTGTGGTAAACACAGAGAAAGGGTATAT | P1066T |
| m3338R | ATATACCCTTTCTCTGTGTTTACCACAACGGCT |  |
| m3759F | AATACAGACAGCACCATTGTGCTCAGGATAATG | P1206H |
| m3759R | CATTATCCTGAGCACAATGGTGCTGTCTGTATT |  |
| m3932F | GGGAAATTGACTGTGTCCCTGTGGCTTG | I1264V |
| m3932R | CAAGCCACAGGGACACAGTCAATTTCCC |  |
| F, forward; R, reverse. | | |

| **Table S3. Plasmid amounts used in the transfection (ng).** | | | | | | |
| --- | --- | --- | --- | --- | --- | --- |
| Group | LEF-LUC | pRL-TK | CMV-LEF | WT or mutant LRP6 | Wnt1 | pcDNA3.1 |
| control | 400 | 20 | 160 | — | — | 420 |
| test | 400 | 20 | 160 | 400 | — | 20 |
| test + Wnt1 | 400 | 20 | 160 | 400 | 20 | — |
| LEF-LUC, luciferase under control of an LEF-1-responsive promoter; pRL-TK, an inner control plasmid expressing Renilla reniformis luciferase; CMV-LEF, plasmid expressing LEF-1 constitutively; WT, wild type; Wnt1, plasmid expressing Wnt1 protein which is agonist of Wnt pathway. | | | | | | |
